# Long-Term Mesh Exposure 5 Years Following Minimally Invasive Total Hysterectomy and Sacrocolpopexy

**DOI:** 10.1007/s00192-024-05769-5

**Published:** 2024-03-26

**Authors:** C. Emi Bretschneider, Erinn R. Myers, Elizabeth J. Geller, Kimberly S. Kenton, Barbara R. Henley, Catherine A. Matthews

**Affiliations:** 1 Division of Female Pelvic Medicine and Reconstructive Surgery, Northwestern University, Chicago, IL USA; 2 Division of Female Pelvic Medicine and Reconstructive Surgery, Atrium Health, Charlotte, NC USA; 3 Division of Female Pelvic Medicine and Reconstructive Surgery, University of North Carolina, Chapel Hill, NC USA; 4 Division of Female Pelvic Medicine and Reconstructive Surgery, University of Chicago, Chicago, IL USA; 5 Division of Female Pelvic Medicine and Reconstructive Surgery, August University Medical Center, Augusta, GA USA; 6 Division of Female Pelvic Medicine and Reconstructive Surgery, Wake Forest University, Winston Salem, NC USA

**Keywords:** Pelvic organ prolapse, Minimally invasive surgery, Mesh complications

## Abstract

**Introduction and Hypothesis:**

The objective was to assess long-term mesh complications following total hysterectomy and sacrocolpopexy.

**Methods:**

In this second extension study, women from a multicenter randomized trial were followed for more than 36 months after surgery. Owing to COVID-19, participants were assessed through either in-person visits or telephone questionnaires. The primary outcome was the incidence of permanent suture or mesh exposure. Secondary outcomes included surgical success and late adverse outcomes.

**Results:**

Out of the 200 initially enrolled participants, 82 women took part in this second extension study. Among them, 46 were in the permanent suture group, and 36 in the delayed absorbable group. The mean follow-up duration was 5.3 years, with the cumulative mesh or suture exposure of 9.9%, involving 18 cases, of which 4 were incident cases. Surgical success after more than 5 years stood at 95%, with few experiencing bothersome bulge symptoms or requiring retreatment. No serious adverse events occurred, including mesh erosion into the bladder or bowel. The most common adverse events were vaginal pain, bleeding, dyspareunia, and stress urinary incontinence, with no significant differences between suture types.

**Conclusion:**

The study found that mesh exposure risk gradually increased over time, reaching nearly 10% after more than 5 years post-surgery, regardless of suture type. However, surgical success remained high, and no delayed serious adverse events were reported.

## Introduction

Pelvic organ prolapse is a complex, chronic condition [1]. For women with advanced uterovaginal prolapse with risk factors for recurrence, surgeons are increasingly adopting abdominal sacrocolpopexy (SCP) for primary surgical repair; however, long-term outcomes, particularly following minimally invasive total hysterectomy and SCP, are sparse [2]. Complicating the clinical picture is the heterogeneity of the surgical technique for minimally invasive SCP, including type of concomitant hysterectomy, suture used for mesh fixation, and graft material. Furthermore, the pathophysiology of mesh-related complications and recurrent prolapse are unclear. Based on the existing literature, it is difficult to ascertain whether adverse outcomes following minimally invasive SCP is related to modifiable factors, such as surgical technique, or related to patient factors, such as connective tissue integrity [3].

This study is the second extension of a randomized control trial that investigated mesh and permanent suture exposure rates following minimally invasive total hysterectomy and SCP. The original study, the Permanent versus delayed-Absorbable Monofilament Suture for Vaginal Graft Attachment during Minimally-Invasive During Total Hysterectomy and Sacrocolpopexy Randomized Control Trial (PACT), randomized participants with advanced uterovaginal prolapse undergoing minimally invasive total hysterectomy and SCP to either permanent suture or absorbable suture for vaginal mesh attachment [4]. One year following the index surgery, the overall rate of mesh or permanent suture exposures was 6.1%, with no difference in mesh-related complications between suture type. In 2022, we reported the findings of the first extension trial of the PACT study (e-PACT), which followed participants for longer than 2 years after the index surgery. At a mean of 3.9 years post-surgery, the cumulative rate of mesh exposure was 7.7% [5].

As the rate of mesh exposure increased between the PACT and e-PACT studies, longer term follow-up beyond 2 years is necessary to better understand factors associated with these adverse outcomes. Thus, the primary objective of this second extension trial of the PACT study, e-PACT II, was to evaluate the rate of long-term mesh or permanent-suture exposure at least 3 years following minimally invasive total hysterectomy and SCP.

## Materials and Methods

This prospective cohort study included women previously enrolled in the multicenter randomized trial, the PACT study [4]. The same five clinical sites that enrolled patients for PACT between April 2015 and May 2019 were involved in this second extension study: Wake Forest Atrium Health, University of North Carolina, Northwestern University, Augusta University, and Atrium Health Carolinas Medical Center. Institutional Review Board approval was obtained at each clinical site. All participants enrolled in the first extension trial (e-PACT) were eligible for the second extension trial, e-PACT II, once 36 months had passed since their index surgery. Participants who did not enroll in e-PACT II but were confirmed to have mesh or permanent suture exposure at any follow-up visit were carried forward as a mesh exposure. The original PACT trial was powered to detect a difference in mesh or permanent suture or mesh exposures between suture type at 1 year; this second extension trial is a prospective cohort trial describing outcomes following minimally invasive total hysterectomy and SCP and was not powered to detect differences in outcomes between suture types.

The primary outcome of this second extension trial was mesh or permanent suture exposure. The outcome measures were defined as examination findings of mesh or permanent exposure through the vagina more than 36 months from the incident surgery. Of note, the IUGA/ICS definition of mesh exposure was adopted in this study: “vaginal mesh visualized through separated vaginal epithelium” [6]. Secondary outcomes included vaginal bleeding, bothersome discharge, partner feeling the mesh, dyspareunia, pelvic pain, and stress urinary incontinence, as well as prolapse treatment success, defined by a composite measure of success:

1. Subjective measures: Pelvic Floor Distress Inventory-20 [7] question 3, response of 2 or higher (i.e., responses of “somewhat,” “moderately,” or “quite a bit” of bother)
2. Objective measures: prolapse recurrence beyond the vaginal introitus
3. Retreatment with vaginal pessary or surgery

Other outcomes of interest included reoperation for mesh exposure. The association of suture type with outcomes was also examined.

For enrollment in e-PACT II, participants were contacted by the following methods: telephone, e-mail, text message, and certified mail. Owing to COVID-19, participants were given the option of an in-person visit or remote assessment of their symptoms at one time point 36 months following surgery. Participants who elected for a remote assessment did not undergo any physical examination, were required to complete several validated questionnaires, and were queried by investigators on protocol-defined postoperative adverse symptoms and events. All participants who reported symptoms suggestive of mesh or suture exposure, including vaginal bleeding, bothersome discharge, and partner feeling the mesh presented for physical examinations. The following validated questionnaires were administered: Pelvic Floor Distress Inventory-20 [7] to assess symptom bother and Patient Global Impression of Improvement (PGI-I) [8] to assess symptom improvement. Protocol-defined postoperative adverse symptoms included vaginal bleeding, discharge, dyspareunia, pelvic pain, partner perception of vaginal mesh exposure, and stress urinary incontinence; postoperative protocol-defined adverse events included any prior assessment or treatment for mesh exposure. The investigators also reviewed the electronic medical record for interim postoperative adverse events. Once the study team received the completed questionnaires from remote participants, participants were sent a Visa Clincard for compensation.

Participants who agreed to an in-person follow-up visit were queried for the same protocol-defined adverse symptoms and events and were required to complete the same validated questionnaires as the remote participants, but also underwent a physical examination, which included a pelvic examination involving a speculum and a bimanual examination, and Pelvic Organ Prolapse Quantification (POP-Q) System assessment [9]. The following were evaluated on examination: permanent suture or mesh exposure, granulation tissue, pelvic pain, vaginal discharge, and vaginal bleeding. Patients were compensated at the completion of the study visit.

All data were entered into a secure database, REDcap, by the research staff at the end of each visit. Study data were monitored for the entire duration of the trial. Means and standard deviations or counts and percentages were computed for univariate analysis. Chi-squared and Student‘s *t* tests were used where appropriate to describe differences in factors between suture type. A *p* value < 0.05 was considered significant. Analyses were performed using R version 3.6.1 (5 July 2019).

## Results

Of the 200 participants enrolled in the original study, 106 participated in the first extension study, and 82 women participated in this second extension study. Of those 82 e-PACT II participants, 56 presented for in-person follow-up whereas 26 participants followed up only via remote assessments. The mean follow-up time was 64 months (5.3 years) after the index surgery, with a range of 36 to 90 months.

Table 1 describes the characteristics of the study cohort. No differences were noted between demographic characteristics between participants who enrolled and those who declined enrollment in the study. Of the participants, 46 were in the permanent suture arm and 36 were in the delayed absorbable suture arm. The mean age was 59 (± standard deviation 9) years, and the mean Body Mass Index (BMI) was 28 kg/m^2^. The majority were white and never-smokers. Nearly 30% were using vaginal estrogen, and the majority of participants demonstrated some degree of vaginal atrophy on examination. No differences were noted in these characteristics across suture type.

**Table 1:** Patient demographics

	Overall (*N* = 82)	Permanent suture (*n* = 46)	Delayed-absorbable suture (*n* = 36)	*p* value
Age, years (SD)	59.4 (9.2)	59.0 (8.8)	59.9 (9.9)	0.64
Body Mass Index, kg/m^2^ (SD)	28.4 (5.4)	29.7 (5.1)	27.6 (5.6)	0.16
Race (%)				0.99
White	75 (91.5)	42 (91.3)	33 (91.7)	
Black	7 (8.5)	4 (8.7)	3 (8.3)	
Smoking history (missing 2) (%)				0.82
Never-smoker	73 (88.8)	37 (82.2)	34 (97.1)	
Former smoker	9 (11.2)	8 (17.8)	1 (2.9)	
Vaginal estrogen (*n* = 79) (%)	23 (29.1)	12 (27.3)	11 (31.4)	0.88
Atrophy (*n* = 56) (%)				0.32
None	18 (32.1)	13 (40.6)	5 (20.8)	
Mild	21 (37.5)	12 (37.5)	9 (37.5)	
Moderate	15 (26.8)	6 (18.8)	9 (37.5)	
Severe	2 (3.2)	1 (3.1)	1 (4.2)	
Months since surgery (median, IQR)	61.5 (44.5, 78.5)	64.0 (56.0, 73.5)	59.0 (54.0, 67.0)	0.11

At a mean follow-up of 64 months (5.3 years), the cumulative mesh or permanent suture exposure from the index surgery was 9.9% (18 cases out of 182 who were eligible for inclusion in the e-PACT trial) with 18 mesh exposures, 3 of which were accompanied by permanent suture exposures. Of those 18 cases, 4 cases (3 mesh exposures and 1 permanent suture exposure) were incident cases since the first extension trial, which followed participants 24 months after the index surgery; thus, the incident rate of mesh or permanent suture exposure between 24 months and>36 months was 4.9% (4 out of 82). In Table 2, the incident mesh or permanent suture-related outcomes between 24 months and >36 months are described. Regarding management of these complications, one of the exposures was expectantly managed, whereas 3 were trimmed in the office; one of these exposures trimmed in the office required a return to the operating room for surgical excision.

**Table 2:** Incident mesh or permanent suture exposures between 24 months and >36 months following surgery

	Overall (*N* = 82)	Permanent suture (*n* = 46)	Delayed-absorbable suture (*n* = 36)	*p* value
Any mesh or suture complication (%)	4 (4.9)	4 (8.7)	0	0.13
Mesh exposure on examination (*n* = 56) (%)	3 (5.4)	3 (9.4)	0	0.35
Suture exposure on examination (*n* = 56) (%)	1 (1.8)	1 (3.1)	0	0.99
Palpable mesh on examination (*n* = 56) (%)	5 (8.9)	3 (9.4)	2 (8.3)	0.99
Granulation tissue (*n* = 56) (%)	1 (1.3)	1 (2.3)	0	0.99
Vaginal bleeding (*n* = 80) (%)	4 (5.0)	3 (6.7)	1 (2.9)	0.80
Discharge (*n* = 76) (%)	1 (1.3)	1 (2.2)	0	0.99

All participants who reported vaginal bleeding or discharge also presented for an in-person evaluation

With regard to other adverse outcomes, a low proportion of participants reported minor complications, such as vaginal bleeding (4 out of 80, 5%), granulation tissue (1 out of 56, 1.8%), or vaginal discharge (2 out of 76, 2.6%). There were no serious adverse events, including no cases of mesh erosion into the bladder or bowel. The most commonly reported adverse symptoms were dyspareunia (6 out of 82, 7.6%), vaginal pain (3 out of 82, 3.9%), and stress urinary incontinence (2 out of 82, 2.6%). The only patients who reported symptoms suggestive of mesh or permanent suture exposure (i.e., vaginal bleeding, bothersome discharge, partner feeling the mesh) were those who had a confirmed mesh exposure on examination; in other words, none of the patients was diagnosed with a mesh or suture exposure by symptoms alone. Table 3 describes the mesh or permanent suture-related exposures for the PACT trial series.

**Table 3:** Description of incident mesh and/or suture complications between surgery and last follow-up

Time frame	Case	Mesh or suture complication	Symptoms	Management
Original PACT trial: up to 12 months following index surgery	1	Mesh	None	None
	2	Mesh	None	None
	3	Mesh	None	None
	4	Mesh	None	None
	5	Mesh	None	Vaginal estrogen
	6	Mesh	None	Vaginal estrogen
	7	Mesh	None	Vaginal estrogen
	8	Mesh	None	Vaginal estrogen
	9	Mesh	None	Vaginal estrogen
	10	Mesh	Vaginal bleeding/discharge	Vaginal estrogen
	11	Mesh and permanent suture	Vaginal bleeding/discharge	Office trimming
	12	Mesh and permanent suture	Vaginal bleeding/discharge	Vaginal estrogen followed by mesh excision in the operating room
PACT extension trial: 12–24 months following index surgery	13	Mesh	Vaginal bleeding/discharge	Office trimming
	14	Mesh	Vaginal bleeding/discharge	Vaginal mesh excision
Second PACT extension trial: 24 to >36 months following index surgery	15	Mesh	Vaginal bleeding/discharge	None
	16	Mesh	Vaginal bleeding/discharge	Office trimming
	17	Mesh	Vaginal bleeding/discharge	Office trimming
	18	Mesh	Vaginal bleeding/discharge	Office trimming followed by mesh excision in the operating room

Of the participants enrolled in e-PACT II, surgical success at a mean of 5 years postoperatively was high. Table 4 describes the outcomes: 2 participants reporting bothersome bulge symptoms, 2 demonstrating prolapse beyond the hymen on examination, 1 undergoing retreatment with pessary; none of the participants underwent retreatment with surgery. The majority of patients reported “Very much better,” “Much better,” or “A little better” on the PGI-I questionnaire (Table 5). No significant differences across suture type were noted in any mesh or permanent suture exposure, treatment success, or other adverse outcomes.

**Table 4:** Prolapse outcomes

	Overall (*N* = 82) (%)	Permanent suture (*n* = 46) (%)	Delayed-absorbable suture (*n* = 36) (%)	*p* value
Composite: treatment success	78 (95)	44 (96)	34 (94)	0.73
Retreatment for pelvic Organ prolapse^a^				
Surgery	0	0	0	NA
Pessary	1 (1.3)	1 (2.2)	0	0.99
Failure based on Pelvic Floor Distress Inventory-20, Item 3^b^	2 (2.5)	1 (2.2)	1 (2.9)	0.99
Failure based on any point beyond the hymen^c^	2 (3.6)	2 (6.3)	0	0.99
POPQ Stage^d^				
2	2 (3.7)	1 (3.1)	1 (4.2)	0.99
3	1 (1.8)	1 (3.1)	0	0.99
4	0	0	0	NA

^a^Total *n* = 78, permanent = 45, delayed absorbable = 33

^b^Total *n* = 80, permanent = 45, delayed absorbable = 35

^c^Total *n* = 56, permanent = 32, delayed absorbable = 24

^d^Total *n* = 56, permanent = 32, delayed absorbable = 24

**Table 5:** Patient Global Impression of Improvement Questionnaire

	Overall *N* = 82 (%)	Permanent suture (*n* = 46) (%)	Delayed absorbable suture (*n* = 36) (%)	*p* value
1: Very much better	61 (73.5)	32 (69.6)	28 (77.8)	0.58
2: Much better	17 (20.5)	11 (23.9)	6 (16.7)	
3: A little better	1 (1.2)	1 (2.2)	0 (0)	
4: No change	0	0 (0)	0 (0)	
5: Much worse	3 (3.6)	1 (2.2)	2 (5.6)	
6L Very much worse	1 (1.2)	1 (2.2)	0 (0)	

## Discussion

In this planned longitudinal cohort study, we demonstrate that women undergoing a minimally invasive concomitant total hysterectomy and sacrocolpopexy with a lightweight polypropylene mesh experience a slow and persistent rise in the rate of mesh exposure from 6.6% at 1 year to at least 10% in the next 4 years. This highlights the critical importance of long-term follow-up of clinical trials for accurate assessment of adverse events.

Although mesh exposure rates in our study are similar to those reported in the extended Colpopexy And urinary Reduction Efforts (CARE) trial (10.5%) [10], the management of vaginal mesh exposure with a lightweight, type 1 polypropylene mesh were notably different, as most exposures in the PACT series were asymptomatic and managed with topical vaginal estrogen alone. Although the rate of mesh exposure was higher than expected, the rate of reoperation for mesh complications in participants who chose to follow up was low, as only 3 women (1.5%) required transvaginal mesh excision surgery: 1 in the 1st year after surgery, 1 between 12 and 24 months, and 1 between 24 months and >36 months. In contrast, 65% of mesh exposures in the extended CARE (e-CARE) trial required surgical excision, which is likely a reflection of the enhanced inflammatory reaction elicited from heavier weight mesh [11].

Mesh exposure rates are impacted by a variety of factors, including colpotomy, route of hysterectomy, and mesh type and weight. The study’s rate of mesh exposure at the time of minimally invasive hysterectomy and SCP was not as high as in older studies that reported mesh exposure rates of greater than 20% following total hysterectomy at the time of SCP [12, 13]. Newer data using lightweight and ultralightweight meshes report no or minimal increase in mesh exposure at the time of hysterectomy [14]. We recently published short-term (up to 1-year) outcomes comparing mesh exposure rates in over 400 women who underwent minimally invasive SCP with either a total hysterectomy or a supracervical hysterectomy using “Upsylon” lightweight (25 gm/m^2^), *n* = 203, or “Restorelle” ultralightweight (18 gm/m^2^) polypropylene mesh, *n* = 200 [14]. Mesh exposure rate at 1 year was 1.5% and did not differ between total and supracervical hysterectomy groups. Also, no mesh exposures were noted in the ultralightweight mesh group, suggesting that mesh weight might be a greater risk factor for mesh exposure at the time of hysterectomy than removing the cervix. Of note, the mesh used in the PACT study was lightweight rather than ultra-lightweight, which may have influenced our results [15].

The PACT study series adds to the outcome data provided by the CARE trial. Until the PACT study, the CARE trial was one of the only randomized trials that reported on prolapse outcomes and mesh-related outcomes following abdominal SCP [10]. There are several differences between the study design of CARE and PACT, which limits direct comparison between the two studies. First, CARE had a mix of participants with post-hysterectomy prolapse and uterovaginal prolapse. Second, in PACT, surgery was performed via a minimally invasive route whereas in CARE the approach was open abdominal. Third, in PACT, total rather than supracervical hysterectomy was performed. Fourth, in CARE, patients were randomized to undergo a concurrent Burch procedure, which can augment the vaginal support. Last the types of mesh used in CARE to support the vagina differed from the lightweight polypropylene mesh used in PACT. Compared with e-CARE, however, our outcomes suggest a better composite prolapse success rate [11]. The probability through year 7 of composite prolapse failure in e-CARE was estimated to be 0.48 for participants who received a Burch and SCP and 0.34 who received an SCP alone. The differences in these outcomes may be because in the PACT study, a total hysterectomy was performed, and data support that retention of the cervix may increase the risk of prolapse recurrence, although it may be protective for mesh-related complications.

Our findings suggest that minimally invasive hysterectomy and SCP might be a safe and effective primary treatment of advanced uterovaginal prolapse. First, no major late adverse events, such as mesh erosions into the bladder or bowel or bowel obstructions, were noted in e-PACT II. Second, the study participants reported high composite surgical success, a finding that mirrors the 2020 study by Culligan et al., which reported a composite surgical success rate of 89% in a retrospective review of outcomes following minimally invasive SCP using ultralightweight mesh with a mean follow up time of 66 months [16]. In the context of our long-term mesh exposure data, we feel that it is relevant to consider the individual risk-to-benefit ratio of prolapse recurrence versus mesh complication in making a recommendation for total hysterectomy and SCP as a primary procedure for prolapse repair.

There are several strengths of this study, including the prospective data collection and use of validated questionnaires for patient outcomes. This study is one of the only studies that provides data on mesh and permanent suture exposure in women undergoing minimally invasive total hysterectomy and SCP with a follow-up of 5 years. Limitations include the loss of participant enrollment and the low number of participants who presented for in-person follow-up given the ongoing COVID pandemic at the time. Owing to this attrition, we may not be capturing patients with mesh or suture exposures, both asymptomatic or symptomatic, as they may be receiving care outside the participating institutions. We are also not able to accurately comment on the success of the surgical outcome given that only 56 of the original cohort presented for an in-person evaluation. The strategy to include patients via a remote visit rather than an in-person evaluation was employed owing to COVID-era restrictions, and we may have missed important physical findings in the cadre of participants who chose remote follow-up. Last, the generalizability of our findings may be limited by the demographic homogeneity of the study cohort.

In summary, minimally invasive total hysterectomy and SCP with the use of a lightweight polypropylene y-mesh is associated with a slowly rising rate of mesh exposure over time. The majority of mesh exposures captured in this study, however, were asymptomatic and managed conservatively. Of the participants who enrolled in this second extension trial, surgical outcomes were excellent with low numbers of recurrence and retreatment.

## Data Availability

Data is available upon request from camatthe@wakehealth.edu.
